# MaLiAmPi enables generalizable and taxonomy-independent microbiome features from technically diverse 16S-based microbiome studies

**DOI:** 10.1016/j.crmeth.2023.100639

**Published:** 2023-11-07

**Authors:** Samuel S. Minot, Bailey Garb, Alennie Roldan, Alice S. Tang, Tomiko T. Oskotsky, Christopher Rosenthal, Noah G. Hoffman, Marina Sirota, Jonathan L. Golob

**Affiliations:** 1 Data Core, Fred Hutchinson Cancer Center, Seattle, WA, USA; 2 Bioinformatics Graduate Program, University of Michigan, Ann Arbor, MI, USA; 3 Bakar Computational Health Sciences Institute, University of California San Francisco, San Francisco, CA, USA; 4 Department of Pediatrics, University of California San Francisco, San Francisco, CA, USA; 5 Department of Laboratory Medicine and Pathology, University of Washington, Seattle, WA, USA; 6 Division of Infectious Disease, Department of Internal Medicine, University of Michigan, Ann Arbor, MI, USA

**Keywords:** microbiome, 16S, machine learning, artificial intelligence, composition, association, phylogenetic placement, phylotype

## Abstract

For studies using microbiome data, the ability to robustly combine data from technically and biologically distinct microbiome studies is a crucial means of supporting more robust and clinically relevant inferences. Formidable technical challenges arise when attempting to combine data from technically diverse 16S rRNA gene variable region amplicon sequencing (16S) studies. Closed operational taxonomic units and taxonomy are criticized as being heavily dependent upon reference sets and with limited precision relative to the underlying biology. Phylogenetic placement has been demonstrated to be a promising taxonomy-free manner of harmonizing microbiome data, but it has lacked a validated count-based feature suitable for use in machine learning and association studies. Here we introduce a phylogenetic-placement-based, taxonomy-independent, compositional feature of microbiota: phylotypes. Phylotypes were predictive of clinical outcomes such as obesity or pre-term birth on technically diverse independent validation sets harmonized post hoc. Thus, phylotypes enable the rigorous cross-validation of 16S-based clinical prognostic models and associative microbiome studies.

## Highlights

• Phylotypes are compositional features generated from 16S-based microbiome studies
• Phylotypes can be more finely grained than species-level taxons
• We develop a software suite to enable phylotype-based analysis
• We demonstrate robust predictive modeling with this approach

## Motivation

A gold standard for statistical power and generalizability of microbiome research is to analyze large datasets representing heterogeneous populations, which can be accomplished by combining data from multiple studies. For 16S rRNA gene variable region amplicon-based microbiome studies, hundreds of thousands of already sequenced specimens are available from public repositories offering an opportunity to achieve this gold standard, but the use of these data is hampered by formidable technical challenges when combining data from technically diverse studies. To overcome these challenges, we developed phylotypes: a taxonomy-independent, stable compositional feature that is generalizable across technically diverse microbiome studies.

## Graphical abstract

**Figure:**
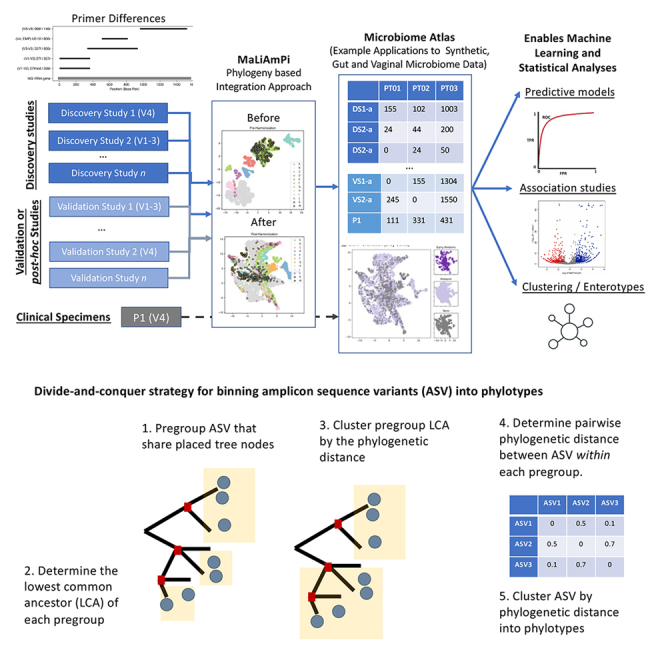

## Teaser

Minot et al. develop a method for the generation of phylotypes, a phylogenetic placement-based, taxonomy-independent compositional feature of microbiota, from technically diverse 16S rRNA gene-based microbiome studies. Phylotypes are biologically meaningful and suitable for clustering, association, and predictive modeling studies.

## Introduction

With the development of high-throughput sequencing, a myriad of studies have associated the human microbiome (the collection of microbes that live within and upon us) with health and disease.^1^^,^^2^^,^^3^^,^^4^^,^^5^^,^^6^ As of 2023, at least 2,000 BioProjects in the NCBI sequence read archive (SRA) contain human microbiome data spanning over 150,000 individual specimens. Owing to challenges with recruiting and retention, microbiome studies are often conducted at a single center and with limited numbers of participants. A complication has arisen as a result: studies of how the microbiome relates to the same biological process frequently report different microbe-disease associations.^7^ For example, multiple studies have associated the human gut microbiome with the efficacy of immune checkpoint inhibitor therapy, with each study finding a different set of bacterial species that associate with a response.^8^^,^^9^^,^^10^^,^^11^^,^^12^ A similar challenge has arisen with the vaginal microbiome adverse pregnancy outcomes such as recurrent pregnancy loss and pre-term birth.^13^ This has limited the translation of microbiome science to clinical practice. The inconsistency of smaller single-center studies is not a unique problem for microbiome studies; similar challenges exist for studies associating with transcription, genetics, and epigenetics. With those ’omics studies, meta-analysis by combining raw data at the sequence or feature level can overcome the challenges of small and single-center studies.^14^ However, a fundamental technical challenge has hampered the combination of microbiome studies, particularly those that target the 16S rRNA gene.^6^

The dominant technique (at least historically) in microbiome science has been amplicon sequencing of a hypervariable region of a taxonomically informative gene such as the 16S rRNA gene. There are nine hypervariable regions in the 16S rRNA gene, each of a size suitable for current high-throughput sequencing platforms. The 16S meta-analysis challenge arises when studies target different variable regions, or even the same variable regions but with differences in the PCR primers, PCR conditions, sequencing library preparation, and the sequencer itself. These technical differences result in the same underlying allele being reported as a different amplicon sequence variant (ASV) and thus not able to be directly combined and compared. This results in specimen-ASV-count matrices from technically diverse studies having little or no overlap. Thus, some harmonization must occur to convert observations of individual sequences or inferred sequences (i.e., ASVs) into a set of compositional features (e.g., specimen-feature-count) that are comparable across studies. Specifically for machine learning (ML)/artificial intelligence (AI) approaches a count matrix such as these are a key input, complementary to other inputs such as estimated alpha diversity and pairwise distance between communities.

Several approaches have emerged for binning reads, generally relying upon some outside reference database. The dominant approaches include closed reference operational taxonomic units (cOTU) and projection to taxonomy (e.g., quantifying each family of microbes present). In cOTU generation each experimentally derived amplicon sequence is aligned against a reference database of full-length 16S sequences.^15^ This technique is highly dependent on how well matched the reference is to the microbial communities being studied. Amplicon sequences without a good matching reference end up lost in this approach. Likewise, some amplicon sequences can have multiple nearly identically scored alignments to reference sequences, particularly when a very broad reference set is used. Adjudicating those nearly identically scoring alignments is a difficult challenge and can lead to sequences from the same underlying true 16S rRNA allele being assigned randomly (and thus ambiguously) to different reference sequences, or being lost. Annotation of 16S rRNA gene variable region sequence variants with taxonomy, followed by grouping read counts at a selected (often family or higher) taxonomic level, is a common tactic (e.g., Pinart et al.,^16^^,^^17^ Chen et al.^16^^,^^17^). Taxonomic assignments to variable region amplicon sequences are limited by the generally poor reliability of taxonomic assignments at more granular ranks (e.g., species or strain), if reasonable down to genus level.^18^^,^^19^

Phylogenetic placement has been previously demonstrated as an effective approach for estimating the relationship (pairwise distance) between microbial communities even from technically diverse studies.^20^ Phylogenetic placement methods for ASVs “place” sequences onto an existing phylogenetic tree,^21^ thereby mapping sequence observations onto tree-derived features such as specific edges of the tree graph. These methods have a number of advantages. Robust methods are available for accommodating and expressing uncertainty deriving from sequence variation.^22^ The feature hierarchy is derived explicitly from relevant sequence data, in contrast to a taxonomy, which may either be discordant with sequence-based relationships or define categories that are indistinguishable using available sequence data. As noted above, phylogenetic placement is an effective means of estimating the pairwise distance between communities from independent and technically diverse studies. Taxonomy-independent compositional features (suitable for generation of a specimen-feature-count matrix) have been derived from individual studies via phylogenetic placement^23^ but have not been thoroughly validated as a means of generating compositional features generalizable across technically diverse studies, nor for harmonization of new data into an existing set of compositional features.

Here, we present an advancement in the technical implementation of phylogenetic placement for harmonizing 16S rRNA gene-based microbiome studies, hypothesizing that granular bins of ASVs could be defined via phylogenetic placement even when the amplicons are generated from primers targeting distinct variable regions and employing different sequencing platforms. We demonstrate a technique that bins ASVs into phylogenetically related groups of sequences after placement onto a common phylogenetic tree of full-length, non-clustered, 16S rRNA alleles to generate taxonomy-independent “phylotype” counts that are finer grained in specificity than species while remaining broadly represented across studies. Further, the technique can successfully integrate ASVs post hoc into an existing set of phylotypes, as is required for validation or clinical deployment of predictive models. With this approach we have scaled up to tens of thousands of specimens across various body sites and clinical domains on routinely available computational resources, which has allowed robust applications of association analysis, clustering, and predictive modeling to the data. This technique is available as a portable and reproducible containerized Nextflow workflow (MaLiAmPi; https://github.com/jgolob/maliampi) immediately applicable to meta-analysis of 16S rRNA gene-based microbiome studies as well as clinical translation of extant studies.

## Results

### Binning of 16S rRNA gene variable region amplicon sequence variants via phylogenetic distance

**Figure 1:**
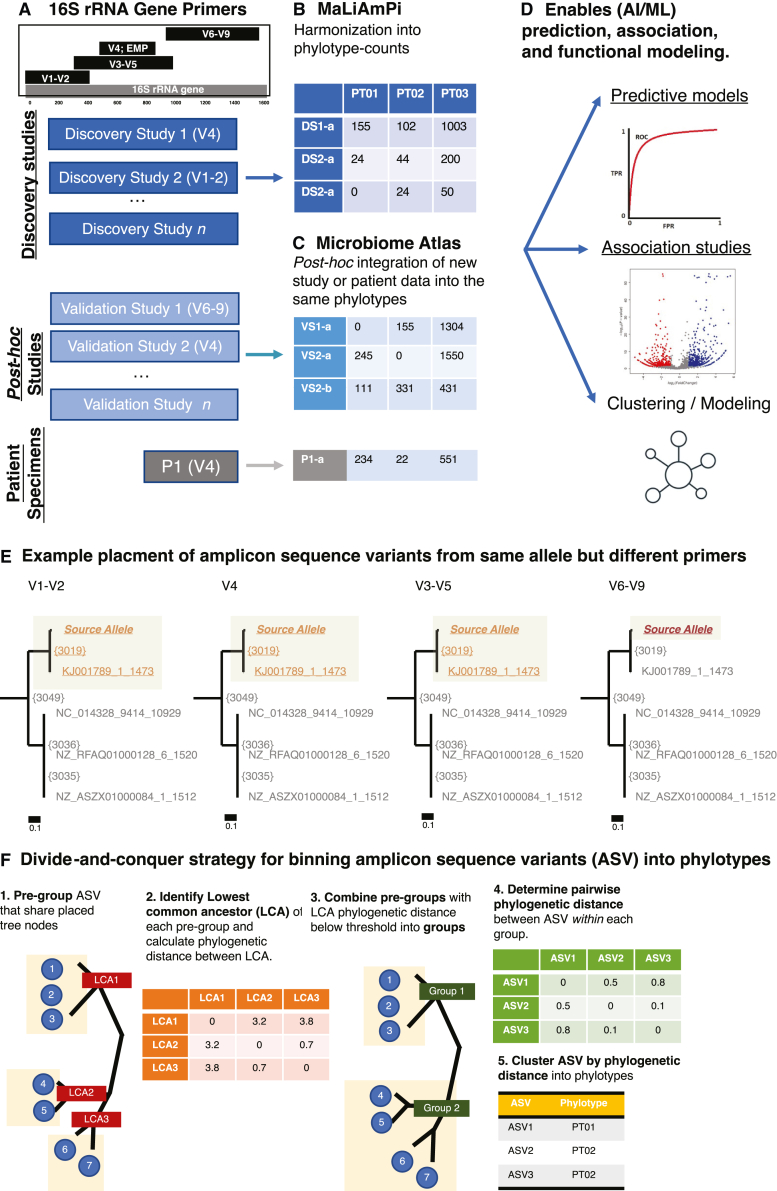
An overview of the challenge of combining data from technically diverse microbiome studies, including the rationale and overall approach (A) Primers targeting four different regions of the 16S rRNA gene, V1-V2, V4, V3-V5, and V6-V9, have largely non-overlapping positions within the full 16S rRNA gene. (B) Depiction of conversion of raw 16S rRNA variable region targeting microbiome studies into one cohesive specimen-phylotype-count matrix. (C) Depiction of post hoc integration of additional 16S rRNA gene variable region amplicon data into an existing set of phylotype features. (D) Depiction of the uses of specimen-phylotype-count matrices for predictive modeling, association and regression, and clustering. (E) A subclade of the phylogenetic tree of full-length 16S rRNA alleles with placement of one amplicon sequence variant (ASV). Distance bar below for phylogenetic distance; the entire subclade is 0.13 phylogenetic distance deep. Leaves of the tree are annotated by the reference sequence and internal nodes by bracketed numbers. Amplicon sequences were generated from the same 16S rRNA allele with primers targeting different variable regions, and thus of distinct and largely non-overlapping sequences (as in A), phylogenetically placed on the tree, with leaves or nodes with likelihood for the given amplicon underlined, gray and non-underlined indicating no likelihood, and increasing saturation of red indicating higher relative likelihood. Despite each ASV having a different sequence, they all phylogenetically place to the same small subclade of the phylogenetic tree that contains the true source allele. The V6-V9 ASV contains sufficient entropy to entirely be placed on the true allele. The lowest common ancestor ({3019}) for all placed nodes or leaves is at a phylogenetic depth of 0.01. (F) Depiction of the divide-and-conquer approach for binning of ASVs phylogenetically placed onto a common phylogenetic tree into phylotypes defined by a phylogenetic distance.

Our objective was to develop a set of taxonomy-independent features that generalize across 16S rRNA gene-based microbiome studies employing different techniques targeting distinct and non-overlapping variable regions of the 16S rRNA gene (Figure 1A) and accurately represent the composition of a microbial community (Figure 1B). Further, this feature set should be able to accommodate new data from future studies (Figure 1C) or clinical patient specimens post hoc, as would be required for validation and practical use of ML or associative studies (Figure 1D).

We considered whether 16S rRNA ASVs from the same underlying 16S rRNA allele but amplified with PCR primers targeting different (non-overlapping) variable regions of the gene would be phylogenetically placed in close proximity. Using *in silico* PCR and employing commonly used 16S rRNA gene variable region primers, we generated ASVs from 100 synthetic human gut-like microbial communities^24^ and then phylogenetically placed these ASVs back onto a *de novo* phylogenetic tree comprising full-length and non-clustered 16S rRNA alleles selected to represent the set of ASVs. As expected, ASVs from the same 16S rRNA allele were placed into the same subpendant of the phylogenetic tree that contained the source allele, often at very short phylogenetic distances from one another, with an example depicted in Figure 1E. Thus, we next focused on binning ASVs by their phylogenetic distance after placement.

The number of ASVs generated from a single microbiome study, let alone ASVs assembled from multiple studies of similar sites targeting different variable regions, can reach into the hundreds of thousands to millions, making an O(n^2^) exhaustive calculation of pairwise phylogenetic distances between ASVs for binning computationally impractical. To address this issue of tractability we developed a divide-and-conquer approach (Figure 1F), first pre-grouping ASVs which have at least one shared node or tip with a likelihood of placement, then combining these pre-groups whose lowest common ancestors were closer together in phylogenetic distance than the specified clustering distance. We only exhaustively calculated the pairwise distances within these groups, then used the pairwise phylogenetic distances to bin ASVs. We consider these phylogenetically binned ASVs to be a set of “phylotypes.”^25^

### Phylogenetic tree selection

Most prior efforts to use phylogenetic placement for harmonization of data across studies have used a modified and branch-length optimized version of the Greengenes phylogeny of 16S rRNA alleles clustered at 99% identity,^20^^,^^23^ specifically version 13.5 that has undergone branch optimization and generalized time reversible rate estimation via RAxMLv8 and modification to be suitable for use in the SEPP placement engine.^26^ Motivated by the observation that for genetically diverse clades of physiologically relevant microbes such as *Prevotella*^27^ or *Gardnerella*^28^ where single-nucleotide polymorphisms (SNPs) in 16S variable regions (lost when clustering at 99% identity) can correspond to functional differences significant to the ultimate host-microbe interaction, we compared placement on the Greengenes phylogeny to placement on a *de novo* phylogenetic tree more tailored to the observed ASVs from the studies to be harmonized. For these *de novo* phylogenetic trees, the tips were composed of full-length (>1,200 bp), high-quality (no more than 1% ambiguous bases) 16S rRNA alleles drawn from NCBI’s NT database which were dereplicated at 100% identity, with an objective of recruiting approximately 10 alleles per ASV. Owing to overlapping matching alleles, this can be satisfied with the order of 10,000 total alleles for typical collections of host-associated microbiota.

**Figure 2:**
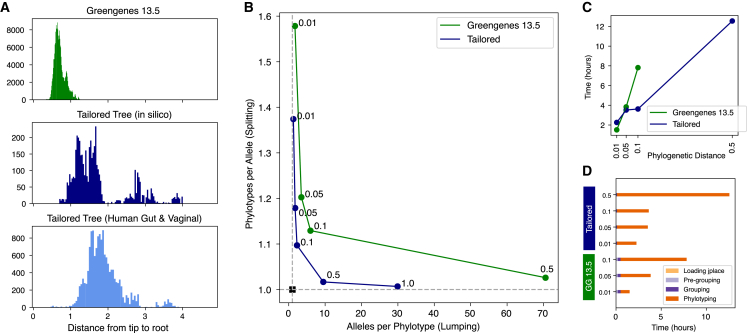
A comparison of phylotypes from the off-the-shelf Greengenes versus custom-tailored *de novo* phylogenetic tree reveals superior performance of the tailored *de novo* tree (A) Tip-to-root distances for the Greengenes 13.5 tree (top), and *de novo* phylogenetic trees generated for ASVs from the *in silico* (middle) or collection of human vaginal and gut microbiota (bottom), with the *de novo* trees comprising tips of full-length non-clustered 16S rRNA SSU alleles. Depicted as a histogram, with the x axis the tip-to-root distance and the y axis the count of tips in that bin of distances. (B) Scatterplot of the mean 16S rRNA SSU gene alleles-per-phylotype (x axis) versus phylotypes-per-allele (y axis) of phylotypes generated after placement on the Greengenes 13.5 phylogeny (green) or *de novo* “tailored” phylogenetic tree (blue) at various binning distances (as labeled next to data points). The ideal outcome is marked as a black square, at one allele per phylotype and one phylotype per allele. (C) Relationship between computational wall time in hours and phylogenetic distance of binning on 87,477 ASVs from six different studies of the human gut microbiome and three of the human vaginal microbiome after placement on two different trees (a tailored tree via MaLiAmPi in navy blue, same as in A; Greengenes 13.5 in green). Testing was conducted on an AMD 5900X CPU, Python 3.10.12 and with Taichi version 1.6.0, llvm 15.0.4. Phylotyping of the Greengenes 13.5 tree at a distance of 0.5 took over 48 h and is not depicted. (D) Computational wall time in hours broken down by the four major steps for phylotyping: loading the placement file, pre-grouping, grouping, and phylotyping. This was for the handling of 87,477 ASVs from six different studies of the human gut microbiome and three of the human vaginal microbiome after placement on two different trees (a tailored tree via MaLiAmPi, same as in A; Greengenes 13.5) at phylogenetic binning distances of 0.01, 0.05, 0.1, and 0.5. Testing was conducted on an AMD 5900X CPU, Python 3.10.12 and with Taichi version 1.6.0, llvm 15.0.4. Phylotyping of the Greengenes 13.5 tree at a distance of 0.5 took over 48 h and is not depicted.

To establish the effect of phylogenetic tree selection on phylotype generation, we compared performance of the off-the-shelf Greengenes 13.5 tree (the most recent available version compatible with placement via SEPP) to ASV-tailored *de novo* phylogenetic trees (via RaXMLv8 with a GTRGAMMA model) on a collection of *in silico* human-gut-like microbiota^18^ amplified with *in silico* PCR with five distinct commonly used variable region targeting primer sets and real-world data from six technically diverse studies of the human gut and three of the human vaginal microbiome (Table S1). The Greengenes 13.5 phylogenetic tree (modified to be suitable for phylogenetic placement in SEPP) has 208,500 tips, whereas the *de novo* phylogenetic trees had 4,644 and 15,331 tips to represent the *in silico* and combined human gut and vaginal ASVs, respectively. The typical tip-to-root depth of the Greengenes phylogenetic tree was shorter than the ASV-tailored *de novo* RAxMLv8 trees, as expected given the very different models and approaches used to generate the trees and the lack of clustering of the underlying sequences for the *de novo* trees (Figure 2A).

We next placed the ASVs onto the trees, using SEPP for the Greengenes phylogeny and pplacer for the tailored *de novo* trees. We then used the resultant placements to bin the ASVs into phylotypes at a range of phylogenetic distances based on the observed typical root-to-tip distances: from 1 to 0.001. We focused our attention on the *in silico* reads, for which we know the “true” source allele of every amplicon. Using this knowledge, we were able to determine the number of alleles-per-phylotype bin (i.e., if there was lumping of different alleles into the same bin) and the phylotype bins per allele (i.e., if there was splitting of ASVs from one allele into multiple phylotype bins) at this range of phylogenetic distances on both trees. An ideal performance would be a 1:1 correspondence between true source allele and phylotype (i.e., neither splitting nor lumping, with one allele per phylotype and one phylotype per allele). As can be seen in Figure 2B, the tailored *de novo* tree binned at a phylogenetic distance of 0.5 or 0.1 came very close to this ideal and closer to any distance for the Greengenes 13.5 tree. Further, the tailored tree demonstrated less sensitivity to the distance parameter with respect to these performance metrics—a desirable characteristic when working with real data (for which we do not know the true origin of each amplicon sequence).

We then established how tree selection and the phylogenetic distance parameter affected memory usage and computational time. Here, we used the placement of 87,477 ASVs from six studies of the human gut and three of the human vagina (Table S1) onto either a custom-tailored tree (as evaluated in Figure 2A for tip-to-root lengths) or the Greengenes 13.5 phylogenetic tree at a range of phylogenetic distances (0.01, 0.05, 0.1, and 0.5). Memory usage scaled largely with the tree size, on the order of a gigabyte of RAM for both trees. Total and subcomponent (tree loading, pre-grouping, grouping, and phylotyping) wall time for computation were determined on an AMD Ryzen CPU (5900X) hosted by Python 3.10.12 and with Taichi version 1.6.0, llvm 15.0.4. As expected, computational wall time had the strongest relationship with the phylogenetic distance selected for binning, with lower values having shorter computational times (Figure 2C); this is due to group sizes being determined by the threshold distance and smaller groups needing fewer pairwise distance determinations. Comparing between trees, the Greengenes 13.5 tree took longer to pre-group and group on (Figure 2D), perhaps reflecting the larger tree requiring additional effort to subset down to the most relevant subclades. Ultimately phylotyping performance was more affected by the phylogenetic distance of clustering than the specific tree selected, with sufficient performance on modest hardware to handle very large sets of ASVs placed onto large trees.

Ultimately, the underlying approach for phylotype generation (as in Figure 1F) is agnostic to the specific placement engine used (e.g., pplacer, epa-ng, or SEPP) and the tree onto which placement is occurring, with the tool able to accommodate ASVs placed onto *de novo* trees from RAxMLv8, RAxML-ng, or the Greengenes off-the-shelf phylogeny. This allows users to use whichever tree they feel is best for their specific collection of studies. Given these results demonstrating a moderate advantage in both parameter sensitivity and overall performance with the tailored *de novo* trees, we proceeded with our *de novo* phylogenetic trees tailored for the ASVs to be harmonized in subsequent studies.

### Phylogenetic-placement-derived phylotypes are generalizable across studies at a finer resolution than species-level taxonomy

The approach of binning ASVs into phylotypes results in a hyperparameter that needs to be selected: phylogenetic distance for clustering. Sequence variants at less than this distance apart will be clustered together to form phylotypes. We established a range of phylogenetic distances (0.1, 0.5, and 1.0) for evaluation, derived from our initial explorations of the typical phylogenetic distance between the placement of ASVs from the same underlying allele (an example depicted in Figure 1E) and when comparing phylogenetic trees (Figure 2B). We applied the phylogenetic placement before binning approach on *in silico* amplicons from 100 human-gut-like communities, as used previously in Golob et al.,^18^ and amplicons from nine real-world studies of human microbiota (six gut and three vaginal) (Table S1). A reference package was generated for each respectively. For the *in silico* data, we compared the same set of 100 communities when amplified *in silico* with primers typically used in 16S rRNA studies. We considered both granularity (how many features that can be derived from a set of specimens) and accuracy (how well these features can represent the “true” underlying microbial communities).

**Figure 3:**
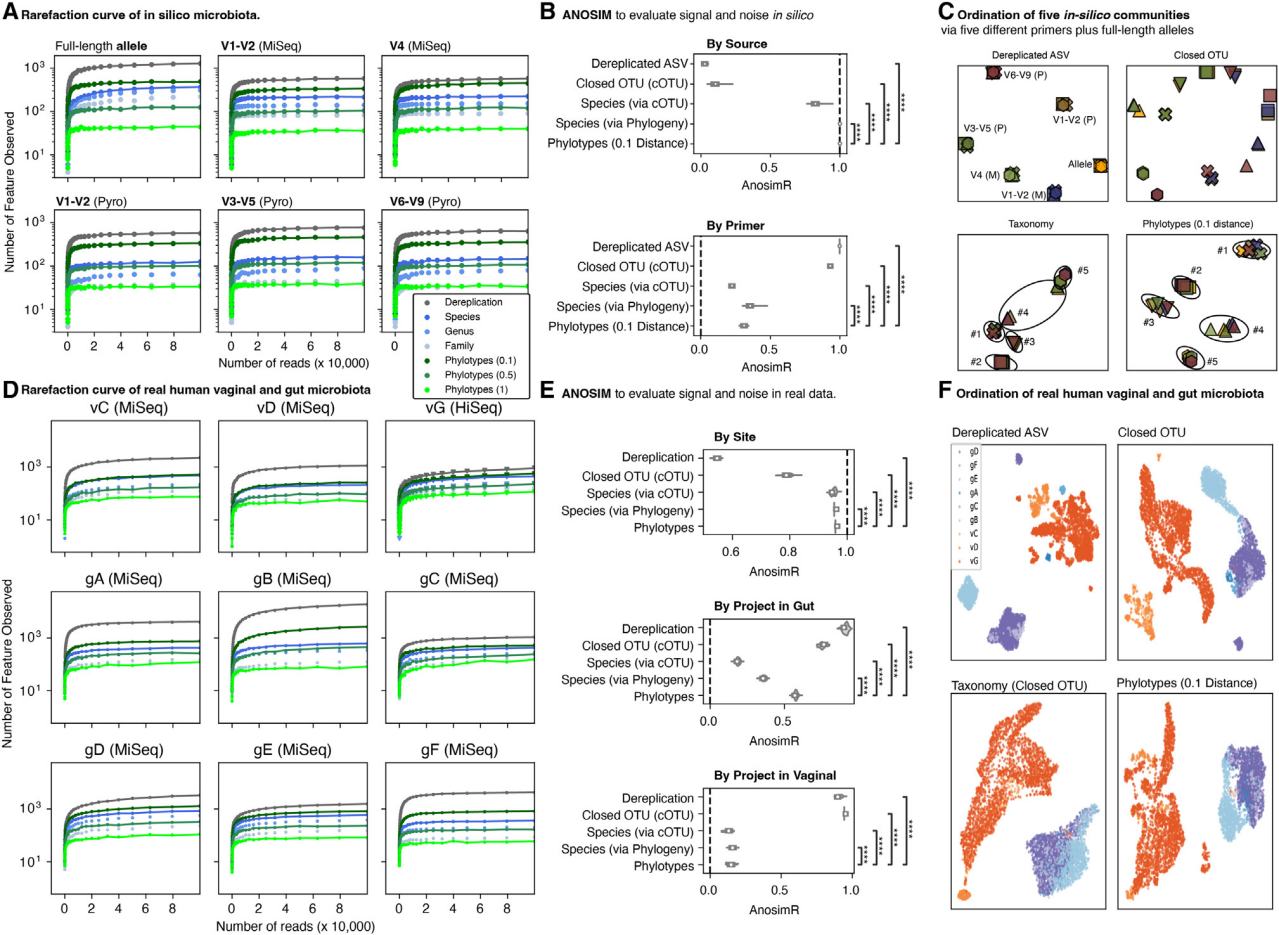
A comparison of phylotypes to taxonomy and closed operational taxonomic units (A) Rarefaction curves of alleles and amplicons derived *in silico* from simulated human gut-like microbial communities. The x axis is number of reads observed, and the y axis is the number of unique features recovered. Gray: raw features, representing the “true” amount of features before clustering, binning, or grouping; green: phylotypes binned at 0.1, 0.5, and 1.0 phylogenetic distance (from darkest to lightest); blue: taxons at species, genus, or family level (from darkest to lightest). (B) ANOSIM regression of pairwise distance versus the “true” source (ideal coefficient 1), or primer used for *in silico* PCR (ideal regression coefficient of 0). (C) Ordination plots generated by UMAP from pairwise Bray-Curtis distance between five simulated human gut-like microbial communities, PCR amplified *in silico* targeting five distinct variable regions (as in Figure 1A) and the full-length alleles, with a different color for each primer (as labeled in the upper left panel) and a different marker for each of the five source communities (as numbered in the lower panels). The raw features were then clustered by dereplication, closed operational taxonomic unit generation (cOTU), taxonomy (species level via cOTU), or phylotypes binned at 0.1 distance. (D) Rarefaction curves of real-world 16S rRNA variable region amplicon data from three studies of the human vagina during pregnancy and six studies of the human gut in health. The x axis is number of reads observed and the y axis the number of unique features recovered. Gray: raw features before any attempt at clustering or binning; green: phylotypes binned at 0.1, 0.5, and 1.0 phylogenetic distance (from darkest to lightest); blue: taxons at species, genus, or family level (from darkest to lightest). (E) ANOSIM regression coefficients with 95% confidence intervals determined by bootstrapping. Real data were regressed against the body site from which the specimen was obtained (ideal coefficient would be 1) or source project stratified by body site (ideal coefficient of zero). (F) Bray-Curtis pairwise distance based UMAP ordination of real data from three studies of the human vaginal microbiome during pregnancy and six studies of the “healthy” human gut. Vaginal studies (vC, vD, and vG) are in orange; gut studies (gA, gB, gC, gD, gE, and gF) are in purple-blue.

For the *in silico* communities, we know the original allele for every amplicon and thus can directly establish both the number of “true” features and the “true” relationships between communities. Rarefaction curves were generated from the source alleles and amplicons generated using primers and read depths typically used in human associated microbiota studies (Figure 3A). Consistently, regardless of primers and sequencing technique simulated, phylotypes at a distance of 0.1 were more granular than species-level taxonomy, with phylotypes at 0.5 distance comparable to genus-level grouping.

We then calculated Bray-Curtis pairwise distances between communities based on pseudo-counts (normalized to 10,000 reads per specimen), including the same communities sequenced with six distinct techniques (five different PCR primers and the full-length alleles). Ideally, the distance between the same community but with a different approach would be zero. We used ANOSIM^29^ to correlate these pairwise distances to the source community across the six distinct techniques (ideally perfectly correlated at 1) or of the same community but with different primers (ideally not correlated, or zero). Phylogenetic placement, whether as species counts or phylotype counts binned at 0.1 distance, were able to retain the best correlation with the community while minimizing the residual correlation with the primer used to amplify (Figure 3B). Uniform manifold approximation and projection (UMAP) ordination based on Bray-Curtis distance of pseudo-counts (normalized to 10,000 reads per specimen) of five randomly selected *in silico* communities (Figure 3C) revealed clustering almost entirely by technique rather than source for dereplicated ASV or cOTU (as expected), and significant lingering overlap between communities with species-level taxonomy. In contrast, phylotype-count-based ordination (binned at a distance of 0.1) cleanly separates by source community into tight groups.

Real-world human vaginal and gut microbiota data rarefaction curves demonstrated that phylotypes at 0.1 distance are more granular than species-level taxonomy across a broad swath of approaches and studies (Figure 3D). Again, we calculated Bray-Curtis distances between the real-world specimens using pseudo-counts (normalized to 10,000 reads). We used ANOSIM to establish the relative strength of “signal” (correlation with body site) to “noise” (correlation with project within the same body site) (Figure 3E). The correlation with body site was the strongest via phylogenetic placement either as species or phylotype counts, much as it was for the *in silico* data, with the phylotype counts marginally superior. Correlation to project was almost entirely removed with species or phylotype counts for the three vaginal microbiome studies. A more interesting pattern emerged for the six gut studies, with phylotype counts having a moderate correlation with the project, perhaps reflecting that the six studies are similar but not biologically identical, and those subtle biological differences are better retained with the phylotype counts. UMAP ordination based on Bray-Curtis distance of feature pseudo-counts revealed the cleanest separation between specimens from different body sites with species or phylotype counts (Figure 3F).

Together, this indicates that phylotypes binned at a distance of 0.1 retain an ability to accurately represent the microbes in a community, are more granular than species, and are generalizable across a broad swath of simulated 16S rRNA amplification and sequencing approaches both *in silico* and with real-world data.

### Phylotype counts are biologically meaningful features for pairwise distance estimation, ordination, and clustering of specimens

**Figure 4:**
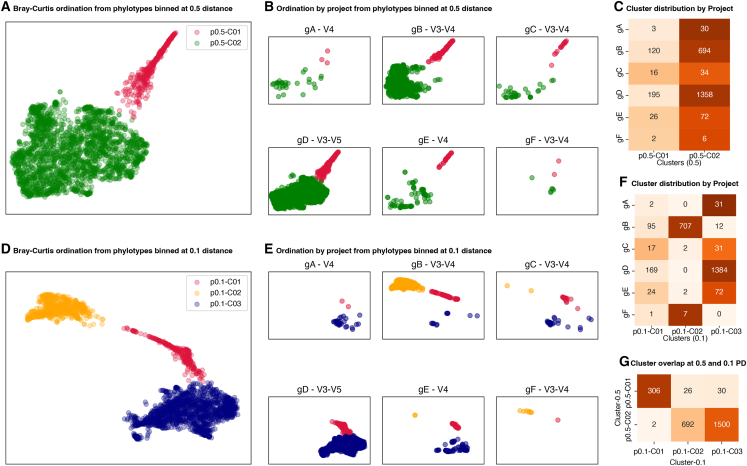
UMAP ordination based on pairwise Bray-Curtis distance of specimens from six independent studies of the healthy human gut clustered by k-means clustering (A) Ordination and two distinct clusters based on phylotype counts when binned at 0.5 phylogenetic distance. (B) Clusters are represented in all six studies in roughly equal proportions. (C) Ordination and three distinct clusters based on phylotype counts when binned at 0.1. (D) Phylogenetic distance. (E) Clusters are represented in multiple studies. (F) Cluster 1 is represented in roughly equal proportion across all six studies, with cluster 2 or 3 predominant in distinct subsets. (G) Cluster 1 from phylotypes at 0.5 phylogenetic distance corresponds to cluster 1 from phylotypes binned at 0.1 phylogenetic distance.

We next focused on the six studies of the “healthy” human gut we were able to separate from vaginal communities (see above). Each has a slightly different definition of healthy and distinct techniques. We built off the prior Bray-Curtis ordination based on phylotype pseudo-counts (normalized to a total of 10,000 reads per specimen) from phylotypes binned at 0.5 and 0.1 phylogenetic distance. At 0.5 phylogenetic distance, the communities cluster into two distinct groups (Figure 4A) represented across all six studies (Figure 4B) in roughly equal proportions (Figure 4C). At 0.1 phylogenetic distance, the specimens cluster into three distinct groups (Figure 4D). The representation (Figure 4E) and proportions (Figure 4F) here do vary across studies, with four of the six studies dominated by specimens in clusters 1 and 3, and two studies with specimens in clusters 1 and 2. Notably, all three clusters are represented in multiple studies (gB, gC, and gE) in a manner that does not clearly track with the variable regions targeted by each study, indicating that these differences are more likely to be biological rather than technical, revealed by the finer disambiguation between organisms at a distance of 0.1 compared to 0.5. This is further supported by cluster 1 being largely overlapping when comparing specimens between 0.1 and 0.5 phylotypes, with cluster 2 at 0.5 distance split into clusters 2 and 3 at 0.1 (Figure 4G).

### Phylotype counts can be biologically meaningful and stable features for regression and machine learning

**Figure 5:**
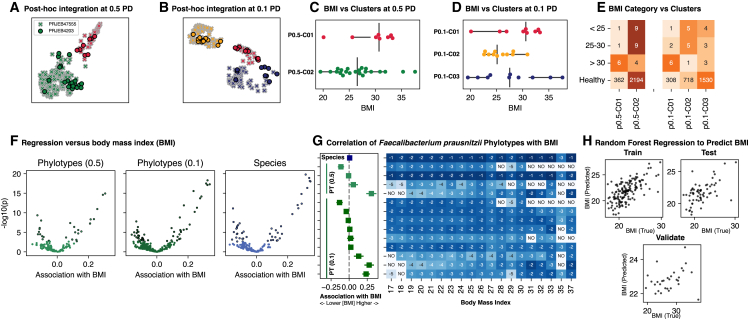
Post hoc integration of gut microbiome studies into an existing phylotype set, ordination, and clusters (A and B) Two independent studies relating body mass index (BMI) and the gut microbiome were harmonized into the existing set of phylotypes and derived UMAP ordination and k-means clusters from phylotypes binned at 0.5 (A) and 0.1 (B) distance. (C and D) SRA: PRJEB4203 BMI stratified by cluster membership based on phylotypes at 0.5 (C) or 0.1 (D) distance. (E) SRA: PRJEB4203 specimens stratified by BMI category, with those with BMI >30 enriched in cluster 1 derived from phylotypes at 0.5 or 0.1 binning distance. (F) Volcano plots of beta-binomial regression of phylotype or species counts versus BMI of specimens from both SRA: PRJEB4203 and SRA: PRJEB47555. (G) A focus on regression results of the species *Faecalibacterium prausnitzii* (Fp) and phylotypes (PT) most likely representing Fp subspecies. The left panel shows the regression coefficients relative to BMI with 95% confidence intervals on the x axis being the regression coefficient with BMI. The 95% confidence interval of the regression coefficient is depicted. The right panel is a heatmap of mean relative abundance of each feature stratified by BMI after log_10_ transformation. Values are the log_10_ order of magnitude. “NO” specifies that this feature was not observed in specimens with this BMI. (H) Random forest regression based on phylotype counts (from binning at both 0.1 and 0.5) and assigned clusters on training and test subsets of SRA: PRJEB47555 as well as validation on specimens from SRA: PRJEB4203 (not used for training or testing).

Next, we integrated post hoc two independent studies of the human gut related to body mass index (BMI) into the already established phylotype sets binned at 0.5 (Figure 5A) and 0.1 (Figure 5B) phylogenetic distance and noted that the specimens from these studies neatly fell into the ordination and clustering scheme previously established. One study (SRA: PRJEB4203) had participants with BMI >30, and the other (SRA: PRJEB47555) was a study of “lean” individuals all with a BMI <30. Over 99% of the reads from these studies could be assigned to a phylotype previously generated. Cluster 1, from phylotypes binned at 0.5 (Figure 5C) or 0.1 (Figure 5D) phylogenetic distance, were enriched with specimens associated with BMI >30 (Figure 5E).

We next completed a beta-binomial regression^30^ of phylotypes or species counts versus BMI and noted a roughly similar pattern of correlation (Figure 5F). Delving into the top ten features within each set that are positively (Table 1) or negatively (Table 2) correlated with BMI, we noted some overlap and one substantive consistency. Thirteen of the top 20 positively associated species had a related phylotype also positively associated with BMI (Table 1), and ten of the top 20 negatively correlated species also had a phylotype negatively associated with BMI (Table 2). The full regression results are available as a supplement for species (Table S2), phylotypes binned at 0.5 (Table S3), and phylotypes binned at 0.1 (Table S4).

**Table 1:** Top 20 features most positively associated with body mass index as determined by Wald T via beta-binomial regression

Species	Phylotype 0.5	Phylotype 0.1
***Dorea formicigenerans***	pt05__01746: a Faecalibacterium prausnitzii	**pt01__00105**: **a Blautia wexlerae/Blautia provencensis**
***Ruminococcus faecis***	**pt05__00227**: **a Xylanivirga thermophila**	pt01__02297: a Blautia phocaeensis/Blautia faecis
***Blautia provencensis/Blautia wexlerae***	pt05__01926: a Oscillibacter ruminantium	pt01__00160: a Fusicatenibacter saccharivorans
***Anaerocolumna cellulosilytica/aminovalerica***	pt05__00134: a Catenibacterium mitsuokai	**pt01__11220**: **a Anaerocolumna cellulosilytica/aminovalerica**
*Marseillibacter massiliensis*	pt05__01928: a Ruminococcus bromii/[Clostridium] viride/Paludicola psychrotolerans	pt01__00127: a Faecalibacterium prausnitzii
***Eubacterium coprostanoligenes***	**pt05__00001**: **a** [**Ruminococcus**] **torques**	pt01__00261: a Fusicatenibacter saccharivorans
***Xylanivirga thermophila***	pt05__01922: a Tyzzerella nexilis/Coprococcus phoceensis	pt01__00089: a Faecalibacterium prausnitzii
***Monoglobus pectinilyticus***	pt05__00042: a Prevotella stercorea	**pt01__00358**: **a Dorea formicigenerans**
*Beduinibacterium massiliense*	pt05__00335: a Sporobacter termitidis/Papillibacter cinnamivorans	pt01__00299: a Oscillospira guilliermondii
***Paraprevotella clara***	pt05__00026: a [Clostridium] innocuum	**pt01__00063**: **a Monoglobus pectinilyticus**
***Holdemanella biformis***	pt05__00035: a Senegalimassilia faecalis/Senegalimassilia anaerobia	**pt01__00042**: **a Ruminococcus faecis**
[***Eubacterium***] ***rectale***	pt05__00060: a Collinsella tanakaei	pt01__00024: a Prevotella stercorea
*Collinsella aerofaciens*	pt05__00043: a Paraprevotella xylaniphila	**pt01__00485**: **a** [**Eubacterium**] **rectale**
*Ruthenibacterium lactatiformans*	pt05__00004: a [Eubacterium] rectale	pt01__00321: a Lachnospira eligens
***Dorea longicatena***	pt05__00049: a Romboutsia timonensis/[Clostridium] dakarense/Romboutsia sedimentorum	**pt01__00184**: **a Eubacterium ramulus**
[*Ruminococcus*] *gnavus*	pt05__00023: a [Eubacterium] saphenum/[Eubacterium] brachy	**pt01__00059**: **a Paraprevotella clara**
*Clostridiales* spp.	pt05__00088: a Duncaniella muris	**pt01__00061**: **a Holdemanella biformis**
***Eubacterium ramulus***	pt05__00116: a Butyrivibrio crossotus	**pt01__00135**: **a Dorea longicatena**
[***Ruminococcus***] ***torques***	pt05__00014: a Ruminococcus flavefaciens	pt01__07299: a Duncaniella muris
*Streptococcus thermophilus*	**pt05__00879**: **a Eubacterium coprostanoligenes**	pt01__00030: a Pseudoflavonifractor capillosus

Features are species counts, or phylotype counts binned at 0.5 or 0.1 phylogenetic distance. Phylotypes are decorated by the most similar species; there can be multiple phylotypes for a given species. Species with related phylotypes are in boldface.

**Table 2:** Top 20 features most negatively associated with body mass index as determined by Wald T via beta-binomial regression

Species	Phylotype 0.5	Phylotype 0.1
*Gemmiger* spp.	pt05__00260: a [Clostridium] fimetarium/Fusimonas intestini/Acetatifactor muris	pt01__00065: a Faecalibacterium prausnitzii
*Christensenellaceae* spp.	**pt05__00005**: **a Bacteroides xylanisolvens**	pt01__00306: a Oscillospira guilliermondii
***Lachnospiraceae* spp**.	pt05__00254: a Faecalibacterium prausnitzii	**pt01__00020**: **a Lachnospiraceae spp**.
***Lachnospira* spp**.	pt05__01930: a Candidatus Galacturonibacter soehngenii	**pt01__00289**: **a Lachnoclostridium spp**.
*unclassified Clostridiales* spp.	pt05__00034: a Bifidobacterium pseudocatenulatum	**pt01__00016**: **a Lachnospiraceae spp**.
***Lachnoclostridium* spp**.	**pt05__01618**: **a Ruminococcaceae spp**.	**pt01__00460**: **a Bilophila wadsworthia**
***unclassified Ruminococcaceae* spp**.	pt05__00086: a Oribacterium spp.	**pt01__00152**: **a Bifidobacterium longum**
***Butyrivibrio* spp**.	pt05__00017: a Coprobacter fastidiosus	pt01__00097: a Bacteroides uniformis
***Bilophila wadsworthia***	pt05__00339: a Oscillospira guilliermondii	**pt01__01171**: **a Bacteroides xylanisolvens**
*Roseburia* spp.	pt05__00052: a Desulfovibrio piger	pt01__11376: a Ruminococcaceae spp.
***Bifidobacterium longum***	pt05__00010: a Alistipes timonensis	**pt01__00090**: **a Bacteroides ovatus**
*Marseillibacter* spp.	pt05__00006: a Bacteroides vulgatus	pt01__00767: a Sporobacter termitidis/Papillibacter cinnamivorans
***Ruminococcaceae* spp**.	pt05__00064: a [Clostridium] fimetarium/Lachnobacterium bovis	pt01__02179: a Oscillospira guilliermondii/Sporobacter termitidis
*Eubacterium* spp.	pt05__00033: a Oscillospira guilliermondii/Sporobacter termitidis	**pt01__00104**: **a Lachnospiraceae spp**.
*Blautia faecis*	pt05__00007: a Bacteroides uniformis	**pt01__00039**: **a Bacteroides xylanisolvens**
***Bacteroides ovatus***	pt05__00149: a Sporobacter termitidis/Monoglobus pectinilyticus	pt01__00230: a Faecalibacillus intestinalis
***Bacteroides xylanisolvens***	pt05__00640: a Candidatus Borkfalkia ceftriaxoniphila/Beduinibacterium massiliense	pt01__01874: a Petroclostridium xylanilyticum/Xylanivirga thermophila
*Shigella flexneri*	pt05__00012: a Ruthenibacterium lactatiformans	**pt01__02138**: **a unclassified Ruminococcaceae spp**.
*Alistipes onderdonkii*	pt05__00056: a Anaerobutyricum soehngenii	**pt01__11810**: **a Butyrivibrio spp**.
*Roseburia hominis*	**pt05__00215**: **a Lachnospiraceae spp**.	pt01__07966: an unclassified Clostridiales spp.

Features are species counts, or phylotype counts binned at 0.5 or 0.1 phylogenetic distance. Phylotypes are decorated by the most similar species; there can be multiple phylotypes for a given species. Species with related phylotypes are in boldface.

We noted an interesting pattern in a highly prevalent species within the human gut, *Faecalibacterium prausnitzii* (Fp), where the species was represented by multiple distinct phylotypes which had opposing consistent associations with BMI (Figure 5G). When binned at 0.5 phylogenetic distance, Fp is represented by four phylotypes (found across both studies); binned at 0.1 distance, Fp is represented by nine phylotypes. In contrast to the Fp species count (which was not correlated with BMI), phylotypes at both binning distances were mixed in their association with BMI (Figure 5G). For example, of the four Fp phylotypes at 0.5 binning, one was strongly positively correlated with BMI and another strongly negatively correlated, indicative of Fp subspecies variation relating to BMI in a manner only observable by 16S with the granularity provided by phylotypes.

Finally, a random forest regressor was trained on phylotype counts (binned at both 0.5 and 0.1 phylogenetic distance) and phylotype-based clusters from 70% of the specimens on SRA: PRJEB47555 and on hyperparameters tuned using the remaining 30% of specimens from SRA: PRJEB47555, and validated on specimens from SRA: PRJEB4203. The resultant predictions (Figure 5H) are monotonic with the true BMI (Spearman’s r of 0.4 on the validation cohort).

This is not a comprehensive re-evaluation of the relationship between the human gut microbiome and BMI. Rather, this serves as a technical demonstration of the phylotype-based approach and potential utility of phylotypes as a compositional feature that is stable across technically diverse studies, including when studies are harmonized post hoc. Our companion manuscript (unpublished data), using phylotypes to predict a risk for pre-term birth from vaginal microbiome data from technically diverse studies, is a further technical demonstration of the utility of this approach.

## Discussion

Combining data from multiple studies has proven to be a fruitful way to improve patient care using ’omics data, within the broad conceptual framework of precision medicine. For example, it is now common practice to use genomics data to personalize and optimize cancer treatment regimens, significantly improving outcomes for patients.^31^^,^^32^ Similarly, transcriptional^33^ and epigenomics studies are being combined and revisited with newer ML techniques with an eye toward drug repurposing and personalized medicine. These successes were contingent upon being able to combine data from multiple independent studies to allow for robust cross-validation of any predictions. The clinical translation requires ability to integrate data from an individual patient into the schema of features used for modeling. Facilitating these efforts are a very clear and intrinsically generalizable set of features, such as SNPs (genomics), loci (epigenomics), and genes (transcriptomics). Microbiome studies have lacked such a clear and generalizable underlying feature. cOTUs and taxons have been attempted when integrating microbiome studies, but both have fundamental limits that we have redemonstrated here or in previous studies.^18^ Thus, lack of a robust and generalizable feature has been a core limitation of microbiome science. It has left the field unclear as to how to apply the findings of a microbiome study to other studies of the same clinical question and to an individual patient and use the microbiome as a biomarker (as is done with genomics data in cancer treatment).

Here we demonstrate a practical approach using phylogenetic placement of ASVs from 16S rRNA allele variable regions to overcome differences in technique (such as primer selection, PCR conditions, and sequencing platform), successfully combining data from multiple studies into one cohesive dataset. Previous applications of this technique focused on generating pairwise distances between communities (e.g., Unifrac^34^) or superior taxonomic assignments to ASVs. Ordination and clustering based on pairwise distances has come under increasing critique for being prone to bias during hyperparameter selection^35^ and can miss when the relationship between a microbial community and functional outcome is driven by which microbe is occupying a minor (from relative abundance perspective) but physiologically potent ecological niche. An example is the methanogenic archaea *Methanobrevibacter smithii*,^36^ which colonizes some human gut microbiota at a low relative abundance but can have a potent effect on butyrate production by other microbes within the gut.^37^ Particularly given the advent of novel ML and AI techniques that can identify and discern such complex and contextual relationships, the lack of a validated direct analog to a sequence variant, operational taxonomic unit, or taxon (species, genus, or family) count matrix after phylogenetic placement has left most studies attempting meta-analysis to continue to focus on taxonomy. Our approach to generate taxonomy-independent phylotype counts after phylogenetic placement presented and validated here has broad applicability for microbiome science, supporting efforts to describe relationships between microbes, ecotypes, associations with microbes, and the use of cutting-edge ML and AI methods dependent upon well-regularized data in matrix format. The approach is implemented in a computationally tractable manner and validated here for use in both regression and AI/ML approaches.

Much as we demonstrated here with BMI prediction, the proximate rationale of this study was to support our recent study presenting VMAP (Vaginal Microbiome Atlas during Pregnancy)^38^ and a crowdsourced AI/ML challenge to predict pre-term birth from the aggregated vaginal microbiome dataset,^39^ with training data identified spanning generations of high-throughput sequencing technologies and targeting a mix of non-overlapping variable regions. To judge the challenge, two independent datasets had to be integrated into the same set of compositional features post hoc. Perhaps the strongest demonstration of the value of this approach can be found in the results of this competition.^39^ Participating teams were provided a variety of datasets derived from the vaginal microbiome, including both taxonomy counts (family, genus, and species level) and phylotype counts (binned at 1.0, 0.5, and 0.1 distance). The best-performing models all made use of phylotype counts, with feature permutation revealing that the models relied upon a similar set of phylotypes as critical features for making accurate predictions.

Based on our results, a *de novo* phylogenetic tree comprising non-clustered 16S rRNA alleles tailored to a set of ASVs to be placed and binned may produce a superior representation of the community across technically diverse studies and reduced sensitivity of the performance to the phylogenetic distance of binning hyperparameters. Our purely algorithmic and automated approach for generation of *de novo* phylogenetic trees (implemented in the refpkg.nf module of MaLiAmPi) both facilitates this approach by others and cannot replace the fidelity or quality of carefully curated and hand-tailored phylogenetic trees, such as those made and published by Greengenes.^40^ The use of off-the-shelf phylogenies has major appeals. Establishment of the phylogenetic tree is computationally intensive. Having one shared tree of 16S rRNA alleles aids in comparability. A shared tree can undergo intense curation by experts and is inevitably a better representation of the “true” genetic relationships. However, this may come at a cost of reduced specificity for a given set of microbiota. This includes the necessity of pre-clustering 16S rRNA alleles. For genetically diverse clades of physiologically relevant microbes, such as Preovotella^27^ or Garnerella,^28^ SNPs in 16S variable regions (lost when clustering at 99% identity) can correspond to functional differences significant to the ultimate host-microbe interaction. Similarly, we made an unexpected finding of subspecies-level variation within the Fp species regarding the directionality of BMI association. Thus, there is a possible advantage to generation of a *de novo* phylogenetic tree of full-length, non-clustered, 16S rRNA alleles relevant to a set of sequence variants when attempting predictive modeling. Nevertheless, the use of bespoke phylogenetic trees in our approach, as opposed to the off-the-shelf Greengenes phylogenetic tree, is a complication; phylotypes are only generalizable when generated against the same phylogenetic tree. Regardless, the phylotype-generating approach and implementation is agnostic to the tree used, and performant enough to apply if one wished to use the Greengenes phylogenetic tree (or any other phylogenetic tree). The utility that generates phylotypes from placed sequence variants is available via the Python Package Index and is agnostic to the tree and framework within which the placements are generated. It can be easily integrated as a plugin within the robust and mature Qiita^41^ framework that already makes extensive use of phylogenetic placement.

We believe that phylogenetic normalization of 16S rRNA gene variable region ASVs is a promising approach for harmonizing microbiome data from different studies that significantly outperforms existing techniques such as cOTU generation and taxonomy. The outputs are suitable for both meta-analysis and precision medicine. This approach is fully implemented as a reproducible and portable Nextflow-based workflow that can facilitate future microbiome studies.

### Limitations of the study

Our evaluation of the approach has some fundamental limits. We employed *in silico* simulated data for portions of the analysis that required knowledge of the “true” origin of a given sequence (e.g., Figure 2B). Such *in silico* data can only approximate true microbiota. We attempted to mitigate this limitation by all other analyses being conducted with real-world data from a variety of technically and biologically distinct studies of human microbiota, with the caveat that the “true” relationships in those data can only be approximated. The performance of this approach with less well characterized microbiomes (e.g., environmental, non-human) remains to be established in future studies. How different associative techniques will interact with phylotypes (as studied for taxons by Nearing et al.^42^) remains an open question and opportunity for study. We selected CORNCOB, given its ability to acknowledge and handle data with different per-specimen read depths, but future study of the better associative approaches is required. Likewise, there is an exciting opportunity to establish how phylotypes can supplement and relate to other forms of ’omics data, such as whole-genome shotgun, metabolomics, transcriptomics, among others. We are hopeful that phylotypes will prove an enabling compositional feature for future efforts involving multi-omics integration.

Our technique cannot overcome some fundamental challenges. If the primers selected for the study fail to amplify a critical member of the community, this technique itself cannot infer the presence of those organisms. Studies with different sequence amplicon lengths are likely to vary in their ability to resolve a given organism, as sequence length and variable region selection affect the available entropy used to distinguish between microbes. The lower-read depth of other pyrosequencing-based studies results in a limit-of-detection difference that also cannot be overcome. This limit-of-detection challenge is shared by approaches such as low-read-depth whole-genome shotgun sequencing (WGS). Further, this approach cannot address technical variance introduced by differences in collection and DNA-extraction protocols. This approach also adds a hyperparameter that must be selected: a phylogenetic distance at which to cluster ASVs. WGS is an alternative technique for microbiome studies but with its own set of analytic challenges and opportunities.^43^^,^^44^ The semi-random priming of reads eliminates some but not all of the cross-study comparability problems between studies, as it does not eliminate differences in sequencers, library preparations, and sequencing depths. It also remains unclear how to integrate WGS and 16S rRNA data into one cohesive dataset. Finally, the breadth, annotation quality, and upkeep of references for WGS data lag behind those of 16S rRNA alleles. The integration of 16S rRNA gene data with shotgun metagenomic data is an active and ongoing effort in our group.

## STAR★Methods

### Key resources table

**Table:** 

REAGENT or RESOURCE	SOURCE	IDENTIFIER
**Deposited Data**		

Fecal microbiota of healthy individuals paired with methane measurements. (Coded as study gA in this paper)	This paper. Submitted to SRA in 2020 from the University of Minnesota.	NIH SRA PRJNA607556
16S Data Healthy Human Cohort (Coded as study gB in this paper)	Hans Hauner et al.^45^	NIH SRA PRJNA701859
16S rRNA sequencing data of 50 healthy adult males. (Coded as study gC in this paper)	This paper. Submitted to SRA in 2020 from Tongji Medical College Huazhong University of Science & Technology	NIH SRA PRJNA663047
Association of Host Genome with Intestinal Microbial Composition in a Large Healthy Cohort. (Coded as study gD in this paper)	Alexandra Zhernakova et al.^46^	NIH SRA PRJEB14839
Human mucosa and stool microbiome Targeted loci. (Coded as study gE in this paper)	Patrick Schloss et al.^47^	NIH SRA PRJNA418115
Healthy human gut microbiome from 8 volunteers. (Coded as study gF in this paper)	Didier Raoult et al.^48^	NIH SRA PRJEB31801
Vaginal bacterial dysbiosis increases risk of preterm fetal membrane rupture, funisitis and neonatal sepsis and is adversely effected by erythromycin treatment. (Coded as study vC in this paper)	David A MacIntyre et al.^49^	NIH SRA PRJEB21325
vaginal microbiota composition in early pregnancy. (Coded as study vD in this paper)	David A MacIntyre et al.^50^	NIH SRA PRJEB30642
Replication and Refinement of a Vaginal Microbial Signature of Preterm Birth. (Coded as vG in this paper)	Gregory Buck et al.^51^	NIH SRA PRJNA393472
The gut microbiota of Colombian adults with varying body mass index	This paper. Submitted to SRA in 2015 from the Colorado Center for Microbial Ecology, University of Colorado at Boulder	NIH SRA PRJEB4203
Targeted metagenomic (16S amplicons) of the fecal microbial communities from young healthy lean students from Munich Germany	This paper. Submitted to SRA in 2021 from the Technical University Munich	NIH SRA PRJEB47555
DECARD: CC11 Dataset	David Fredricks et al.^18^	doi.org/10.5281/zenodo.1120360
ARF/YA16Sdb collection of curated 16S rRNA alleles	This paper.	doi.org/10.5281/zenodo.6876634

**Software and Algorithms**		

MaLiAmPi	This paper.	https://github.com/jgolob/maliampi
phylotypes	This paper.	https://github.com/jgolob/phylotypes; https://pypi.org/project/phylotypes/
arf/ya16Sdb	This paper.	https://github.com/jgolob/arf
DADA2	Benjamin Callahan et al.^52^	Docker: quay.io/biocontainers/bioconductor-dada2:1.26.0--r42hc247a5b_0
Dada2-pplacer	This paper	Dockerhub: golob/dada2-pplacer:0.8.0__bcw_0.3.1A
fastcombineseqtab	This paper	Dockerhub: golob/dada2-fast-combineseqtab:0.5.0__1.12.0__BCW_0.3.1
Barcodecop	This paper	Dockerhub: golob/barcodecop:0.5__bc_1
TrimGalore	Felix Krueger	Docker: quay.io/biocontainers/trim-galore:0.6.6–0
Vsearch	Rognes et al.^53^	Docker: quay.io/biocontainers/vsearch:2.22.1--hf1761c0_0
Fastatools	This paper	Dockerhub: golob/fastatools:0.8.0A
Pplacer	Matsen et al.^21^	Dockerhub: golob/pplacer:1.1alpha19rc_BCW_0.3.1A
Infernal	Nawrocki et al.^54^	Docker: quay.io/biocontainers/infernal:1.1.4--h779adbc_0
RAxML-ng	Kozlov et al.^55^	Docker: quay.io/biocontainers/raxml-ng:1.0.3--h32fcf60_0
RAxMLv8	Stamatakis^56^	Docker: quay.io/biocontainers/raxml:8.2.4--h779adbc_4
Taxtastic	This paper.	Dockerhub: golob/taxtastic:0.9.5D https://github.com/fhcrc/taxtastic
Epa-ng	Barbera et al.^57^	Docker: quay.io/biocontainers/epa-ng:0.3.8--h9a82719_1
Gappa	Czech et al.^58^	Docker: quay.io/biocontainers/gappa:0.7.1--h9a82719_1
MaLiAmPi	This Paper	10.5281/zenodo.8329650
Phylotypes	This Paper	10.5281/zenodo.8393203
arf/ya16Sdb	This Paper	10.5281/zenodo.10015301

### Resource availability

#### Lead contact

Further information and requests for resources and reagents should be directed to and will be fulfilled by the lead contact, Jonathan Golob (jonathan@goloblab.org).

#### Materials availability

This study did not generate new unique reagents.

#### Data and code availability

• Please see Table S1 for the publicly available read data, obtained from the NIH NCBI Sequence Read Archive. The in silico datasets used are available via Zenodo, at 10.5281/zenodo.1120360. The set of full-length ref. 16s rRNA alleles (processed by ARF) can be found on Zenodo at https://doi.org/10.5281/zenodo.6876634.
• The core MaLiAmPi workflow is open source (MIT license) available at Github (https://github.com/jgolob/maliampi), with the version tagged v3.5.0 used for this manuscript and available archived at Zenodo at https://doi.org/10.5281/zenodo.8329650. The phylotype generation tool is open source (MIT license) available at Github (https://github.com/jgolob/phylotypes) and installable via pip via the Python Package Index pypi.org (‘pip3 install phylotypes’), with version 1.0.1 used for this manuscript available at https://doi.org/10.5281/zenodo.8393203. ARF is a workflow used to create the repository of full-length 16s rRNA alleles and is open source (MIT license). It is available as a Github repository (https://github.com/jgolob/arf) and at https://doi.org/10.5281/zenodo.10015301.
• Any additional information required to reanalyze the data reported in this paper is available from the lead contact upon request.

### Method details

#### Phylogenetic placement of 16S rRNA gene ASVs via MaLiAmPi

MaLiAmPi (Maximum Likelihood Amplicon Pipeline) is a *Nextflow*-based workflow that implements the approach described in this article. The workflow is 100% containerized and portable, and can be run locally (via Docker), on public clouds (such as Amazon Web Services Batch), or academic high performance computing clusters (e.g., SLURM or Sun Grid Engine-based) via Singularity containers. There are four broad steps MaLiAmPi implements: (1) generation of ASVs; (2) selection of a repository of full-length 16S rRNA alleles; (3) generation of a reference package including a phylogenetic tree of full-length 16S rRNA alleles from the repository that match the ASVs; and (4) placement of the ASVs onto the reference package phylogenetic tree.

#### Generation of amplicon sequence variants (ASVs) from FASTQ files

As noted in the Main section, the overall approach is relatively agnostic to the method used to generate ASVs. MaLiAmPi uses DADA2 by default, based in part on prior benchmarking studies.^59^ For Illumina reads, if index reads are available demultimplexing is confirmed with Barcodecop (version 0.5). Reads are then filtered, trimmed and have residual primer and linker sequences removed with TrimGalore (version 0.6.6–0). Amplicon sequence variants are then generated using DADA2 (version 1.18.0). Reads are grouped into Batches, ideally representing a group of specimens processed into a library together, and typically of a size of 100. Each specimen’s reads (or read pairs) are then filtered and trimmed (in parallel) with DADA2’s filterAndTrim with the following parameters for Illumina reads.

**Table:** 

maxN	0
maxEE	Inf
truncQ	2
trimLeft	0
truncLen	(0, 0)

And with the following parameters for 454/Pyrosequencing reads.

**Table:** 

maxN	0
maxEE	Inf
truncQ	2
trimLeft	0
truncLen	250
maxLen	Inf

Filtered and trimmed reads are then dereplicated with the DADA2’s derepFastq command.

The filtered and trimmed reads are grouped into batches, and then the learnErrors command is used to generate an error model for each batch’s forward (and when available) reverse reads with the following parameters for Illumina data.

**Table:** 

MAX_CONSIST	10
Randomize	TRUE
nbases	1e8

And these parameters for 454/Pyrosequencing data.

**Table:** 

MAX_CONSIST	10
Randomize	TRUE
nbases	1e8
HOMOPOLYMER_GAP_PENALTY	−1
BAND_SIZE	32

By batch, the batch’s error model is applied to the dereplicated reads using the dada command with the pool = "pseudo" option for all data, additionally HOMOPOLYMER_GAP_PENALTY = −1, BAND_SIZE = 32 for 454/pyrosequencing data.

On a per-specimen basis, paired-end reads are merged with the mergePairs command with the following parameters.

**Table:** 

trimOverhang	TRUE
maxMismatch	0
minOverlap	12

The minOverlap parameter occasionally needs to be relaxed down to a lower number depending on the PCR primer design and specific Illumina chemistry used, specifically when most or all read pairs fail to merge. For very-low quality read data (e.g., when read pairs fail to merge even with a min overlap of 4), we will only use the forward read data (as we believe those reads cannot be accurately paired).

Finally the merged read pairs or dada models for unpaired reads are converted to sequence tables with the makeSequenceTable command. From these sequence tables are the ASV sequences and specimen-ASV counts extracted into FASTA and CSV formats respectively for subsequent analysis.

#### Repository sequence selection

We started with the deduplicated -> 1200bp -> filtered -> named subset of 16S rRNA alleles from NCBI via the YA16SDB pipeline as our repository of sequences. As noted in the Main section, other repositories of 16S rRNA alleles can also be employed (e.g., SILVA, RDP, Greengenes, etc). This entire set of YA16SDB reads are available for download (as below in the Data Availability section) on Zenodo (https://doi.org/10.5281/zenodo.6876634). A subset of repository candidate full-length 16S rRNA alleles are identified by searching the repository sequences for matches with at least 80% identity to at least one ASV sequence using vsearch (version 2.17.0) in usearch_global mode, and max_accepts = 10. To ensure the resultant tree will not result in overfitting or over diffusion of ASV placement later, full-length 16S rRNA alleles are recruited from the repository with the objective of having roughly the same number of recruited reference sequences per each amplicon sequence variant. Specifically, we establish the best possible percent identity between each ASV and the repository alleles, and discard any alleles that are below this best possible percent identity (e.g., retain the bounded-best-hits). We then determine how many ASVs each reference is a best hit for and discard those that are not a best hit for at least two ASVs. Finally we backfill references for ASVs that no longer have a reference sequence as good as their best it, focusing on the longest alleles with no ambiguous bases and with a precise taxonomic annotation. Even for very broad sets of ASVs, this typically results in less than 30,000 reference alleles.

#### Reference package recreation

These filtered reference alleles are now aligned with cmalign from the Infernal package using the SSU_rRNA_bacteria covariance matrix from the rfam database and a mxsize 4096. The recruited full-length 16S rRNA alleles alignment is then assembled into a phylogeny. The generation of the phylogenetic tree is the most computationally intensive step in the entire approach. The current implementation default to RAxML (version 8.2.4), but also allows RAxML-ng (1.0.3) to be used if desired for a deeper exploration of possible starting random trees.

For RAxML, the following settings are used.

**Table:** 

-m	GTRGAMMA
-p	12345

And for RAxML-ng.

**Table:** 

model	GTR+G
seed	12345
tree	pars{1},rand{1}
bs-cutoff	0.3

This *de novo* phylogenetic tree is combined with the metadata for each allele within the tree (e.g., species-level taxonomy, source accession, etc) into a standardized reference package format using the taxtastic package.

#### Placement of ASVs onto a reference package phylogenetic tree

ASVs are next placed onto this reference tree. First the ASV sequences are aligned, using cmalign from the Infernal package, and the same covariance matrix as was used to make the alignment of reference sequences (retained in the reference package). The ASV alignment is combined with reference alignment (contained within the reference package) using esl-alimerge utility from easel.

This combined alignment is then used to phylogenetically place the ASVs onto the reference package tree using either pplacer (the current default) or epa-ng. Both have comparable performance and outputs. For pplacer, the following parameters are used.

**Table:** 

-p	
--inform-prior	
--prior-lower	0.01
--map-identity	

For epa-ng.

**Table:** 

--baseball-heur	

For SEPP to place on the Greengenes 13.5 ‘off the shelf’ taxonomic tree, we ran the command:

/sepp-package/run-sepp.sh/working/asv/sv_2022-03-08.fasta gg -x 12.

Within a docker container created based on the sepp-package: golob/sepp-greengenes:4.5.1. In turn, this is based on the gg_13_5_ssu_align_99.fasta alignment and associated tree from Greengenes version 13-5 of alleles clustered at 99% global identity.

The output of the placement step is in JPLACE format, dedup.jplace. For each ASV, the likelihood, distal-length, and pendant-length is reported for each edge in the tree (omitting edges for which there is no meaningful likelihood). These likelihood-weighted trees are the basis for subsequent analysis. Combined with ASV-counts-per-specimen, the weighted tree can be used to estimate pairwise phylogenetic distance (KRD-distance, akin to weighted UniFrac) between specimens, the alpha diversity of a specimen, and to group ASVs into phylotypes. Phylotypes are groups of ASVs clustered at a specific phylogenetic distance, and are created using a Python package (https://github.com/jgolob/phylogroups) installable via pypi (https://pypi.org/project/phylotypes/). A distance of 1 roughly corresponds to a species of bacteria, but with significant variation depending on the degree of taxonomic - phylogenetic concordance.

#### In silico human gut microbiota for validation

As in our prior work,^18^ we used 100 microbial communities similar in structure and composition to those found in the healthy human gut microbiome, but generated *in silico* and thus with a known allele of origin for each and all amplicons generated. These communities are available via Zenodo (10.5281/zenodo.1120359). For each community, we have selected specific full-length unambiguous 16S rRNA gene alleles to represent each microbe within the community. From these alleles we can generate amplicons targeting specific hypervariable regions via *in silico* PCR.

We selected primers targeting the most common variable domains and sequencing platforms represented in the large volume of legacy 16S rRNA gene data available in public repositories. Specifically, the V4 region (or V3-V6), V1-V2, and V5-V9 domains (Figure 1) and the sequencing platforms Illumina MiSeq or Roche 454 (a legacy technology for which SRA contains 139,965 records with the label ‘16S’). For MiSeq we set a goal of 50,000 simulated amplicons per community and for 454 we targeted 5,000 amplicons per community, reflecting the typical read-depths from the respective platforms. As depicted in Figure 1, there is effectively no overlap between the amplicons targeting distinct regions (i.e., no overlap in sequence between the primers targeting V1-V2 and V5, nor with V6-V9).

**Table:** 

Primer Set	Variable Region	Intended Platform	Simulated amplicons per community
27fmod/338r	V1 - V2	Illumina MiSeq	50,000
U515f/806r	V4	Illumina MiSeq	50,000
27f/357r	V1 - V2	454	5,000
357f/926r	V3 - V5	454	5,000
968f/149r	V6 - V9	454	5,000

#### Dereplication of ASVs

ASVs with the exact same sequence (length and each base pair) were combined together and assigned an ID.

#### Generation of closed OTUs

Here we used the QIIME1 package, and the Greengenes 97% OTU repository. We generated a docker container containing QIIME 1 version 1.9.1A, and ran the following commands to generate blast-picked closed OTUs with a similarity of at least 80%:

pick_otus.py -i <raw_fastq> -o blast_picked_otus/ -m blast -r 97_otus.fasta -s 0.8.

Where the 97_otus.fasta were the 97_otus from the Greengenes repository, as recommended by the QIIME1 documentation.

#### Calculation of Bray-Curtis distance

Count tables were first assembled with one row per specimen and one column per feature (dereplicated ASV, closed-OTU, or phylotype) and each cell the number of reads assinged to that feature and specimen. These raw-count tables were then normalized to a read depth of 10,000 reads per specimen. The normalized count tables were then used to calculate pairwise Bray-Curtis distance using the *scipy* (verison 1.6.3) pairwise distance calculator.

#### UMAP ordination

The *Python* umap-learn package (version 0.5.1) was used with the following hyperparameters: min_distance = 0

n_components = 2

n_neighbors = 45.

Random state was fixed at 42. The pre-computed Bray-Curtis distance (as above) was used.

#### Generation of phylotypes

Amplicon sequence variants were then grouped into phylotypes via a utility that accepts the jplace-formatted^60^ placement of the sequence variants onto the full-length 16S rRNA allele phylogeny. Sequence variants are then grouped into clusters via agglomerative clustering using phylogenetic distance,^22^ set to generate clusters at a distance threshold (0.1, 0.5 and 1). To avoid the exhaustive O(n^2^) calculation of KR-distance between all sequence variants, the sequence variants are first partitioned into those with placements on similar subclades of the tree. These partitions are then combined when the distance between the lowest common ancestor of each partition is less than the clustering threshold distance. Within each remaining partition, the calculation of pairwise phylogenetic distance is used for clustering.

Post-hoc integration of novel sequence variants into an existing set of phylotypes requires the placement of the new sequence variants onto the same phylogenetic tree as used to generate the phylotypes, the placements of the ‘seed’ sequence variants used to generate the phylotypes, the binning distance, and the phylotype assignments of the existing sequence variants. The process is to (1) determine the lowest common ancestor of each *existing* group of sequence variants binned into phylotypes; (2) determination of the pairwise phylogenetic distance between the *new* sequence variants to the lowest common ancestors of the *existing*; (3) assignment of the new sequence variant to the phylotype with the lowest pairwise distance that is below the specified threshold. New sequence variants that cannot be assigned by this approach are not assigned to a phylotype and can be made into a new phylotype if needed.

### Quantification and statistical analysis

#### Calculation of Bray-Curtis distance

Count tables were first assembled with one row per specimen and one column per feature (dereplicated ASV, closed-OTU, by-species, or phylotype) and each cell the number of reads assigned to that feature and specimen. These raw-count tables were then normalized to a read depth of 10,000 reads per specimen. The normalized count tables were then used to calculate pairwise Bray-Curtis distance using the *scipy* (verison 1.6.3) pairwise distance calculator.

#### Random forest regression

The RandomForestRegressor module of the Python scikit-learn package was used with the following hyperparameters: n_estimators = 2300, criterion = 'poisson', max_depth = 6, min_samples_split = 2, min_samples_leaf = 1, min_weight_fraction_leaf = 0.0, max_features = None, max_leaf_nodes = None, min_impurity_decrease = 0.0, bootstrap = True, oob_score = False, random_state = 42, verbose = 0, ccp_alpha = 0.0, max_samples = 0.05. The fit data included valencia community state types, and phylotype as present/absent at both 0.1 and 0.5 phylogenetic binning distances.

## Supplemental information

Table S1. NIH SRA BioProject IDs for publicly available human vaginal and gut microbiome data used in this study As per the STAR Methods section “data and code availability” under “resource availability”.

Table S2. CORNCOB regression results of species versus body mass index, related to Tables 1 and 2

Table S3. CORNCOB regression results of phylotypes at 0.5 distance versus body mass index, related to Tables 1 and 2

Table S4. CORNCOB regression results of phylotypes at 0.1 distance versus body mass index, related to Tables 1 and 2
